# Diagnostic Challenges of Systemic Lupus Erythematosus

**DOI:** 10.7759/cureus.50132

**Published:** 2023-12-07

**Authors:** Ana Correia de Sá, Marta Batista, Ana L Ferreira, Daniela Casanova, Bebiana Faria, Jorge Cotter

**Affiliations:** 1 Internal Medicine, Hospital da Senhora da Oliveira, Guimarães, PRT; 2 Cardiology, Hospital da Senhora da Oliveira, Guimarães, PRT

**Keywords:** pre-eclampsia, hypertension, proteinuria, libman-sacks endocarditis, antiphospholipid syndrome, systemic lupus erythematosus

## Abstract

Systemic lupus erythematosus (SLE) is a disease characterized by clinical heterogeneity with unpredictable course. Several disease endotypes have been identified, including SLE with antiphospholipid syndrome (APS). We report a case of a pregnant woman with hypertension and proteinuria, diagnosed with APS, Libman-Sacks endocarditis that led to moderate to severe mitral valve insufficiency, and SLE. We describe the diagnostic steps, evolution, and complications. This case highlights the asynchrony behavior of SLE, emphasizing the importance of a multidisciplinary approach to an early diagnosis.

## Introduction

Systemic lupus erythematosus (SLE) is a systemic disease that can affect specific organs, presenting with clinical heterogeneity and unpredictable course, which can pose diagnostic challenges. SLE has several endotypes, such as SLE with antiphospholipid syndrome (APS) [1,2]. APS is an autoimmune disease, characterized by the presence of antiphospholipid antibodies, causing venous or arterial thrombosis, and can be associated with pregnancy morbidity [3-5]. Both diseases are multisystemic, and they can involve rare conditions such as Libman-Sacks endocarditis. Libman-Sacks endocarditis is a rare, non-infectious form of endocarditis characterized by the deposition of sterile platelet thrombi on heart valves. It can be associated with severe valvular dysfunction, sometimes requiring surgical replacement [6,7]. Patients with SLE with concomitant APS represent a complex endotype of the lupus spectrum [2].

## Case presentation

A young Caucasian woman presented for an Internal Medicine consultation in April of 2021. Her history began two years earlier, when she was a 30-year-old woman in the thirty-second week of pregnancy, with fetal growth restriction. She presented to the emergency department with elevated blood pressure. Her past medical history was unremarkable, it was her first pregnancy, and she didn't have any fertility-related complications. She denied nausea, vomiting, headaches, syncope, dizziness, or pain. On examination in the emergency department, her blood pressure was 150/100 mmHg, with no other notable findings. Blood analysis highlighted a creatinine level of 1.24 mg/dL, hemoglobin of 11.8 g/dL, and urine protein/creatinine ratio of 0.78 mg/mg. Platelet, bilirubin, and transaminase levels were within the reference ranges.

The patient was admitted to the obstetrics department with a diagnosis of pre-eclampsia. The patient remained asymptomatic, with blood pressure consistently below 140/90 mmHg, without antihypertensive treatment. The highest value of creatinine was 1.4 mg/dL, and a 24-hour urine analysis demonstrated 1230 mg of proteinuria. After eight days, the patient was discharged without symptoms and no need for antihypertensive drugs.

In the thirty-sixth week of pregnancy, she was readmitted for labor induction and underwent a cesarean section due to a non-reassuring fetal status during labor. The patient and the newborn exhibited a favorable evolution. The mother presented a systolic blood pressure between 135 and 140 mmHg and a diastolic blood pressure between 80 and 95 mmHg. She was discharged on daily 30 mg of nifedipine and referred to an Internal Medicine consultation. At this time, the creatinine value was 1.7 mg/dL and no protein/creatinine ratio was available for consultation.

One month after labor, the patient reported symptoms consistent with Raynaud's phenomenon. Physical examination revealed erythema on the hands and malar area. Blood analysis revealed triple-positive antiphospholipid antibodies: positive lupus anticoagulant, immunoglobulin G (IgG) antibody anti-cardiolipin of 108 U/mL (reference value <10), and IgG antibody anti-β2-glycoprotein-I of 198 GPL (reference value <10), with normal ranges of IgM.

**Figure 1:**
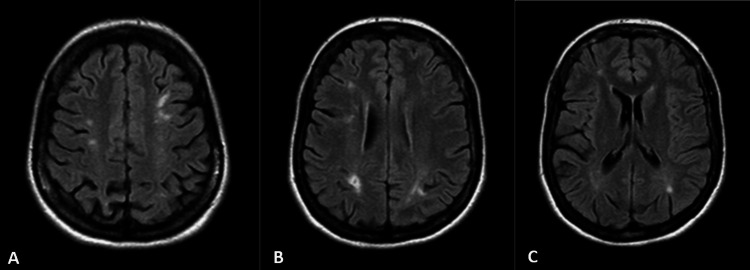
Corticosubcortical lesions in brain magnetic resonance angiography (A, B, and C)

Due to the coronavirus disease 2019 (COVID-19) pandemic, the patient was only able to return for her first Internal Medicine consultation 18 months later. At that time, she was taking no medication. She decided to stop the antihypertensive drugs, as her blood pressure was persistently below 130/70 mmHg. The erythema on her hands and malar area persisted. Upon reviewing her clinical history, she presented a high-risk profile of antiphospholipid antibodies, meeting the criteria for triple-positive, with persistently elevated antibody levels (positive lupus anticoagulant, IgG antibody anti-cardiolipin of 112 U/mL, and IgG antibody anti-β2-glycoprotein-I of 162 GPL). Her creatinine level was 1.2 mg/dL, and the protein/creatinine ratio was 0.34 mg/mg. The patient was started on daily 100 mg of acetylsalicylic acid. By that time, we noted that a head computed tomography performed a few months earlier, after a car crash, revealed hypodense lesions on both sides of the parietal white matter, especially on the left. Brain magnetic resonance angiography showed cortico-subcortical lesion areas with hypersignal on long TR sequences and discreet hyposignal on T1, in the frontal and parietal regions bilaterally. These lesions did not show diffusion restriction or contrast enhancement but were associated with mild accentuation of adjacent sulci, suggesting chronic ischemic lesions (Figure 1).

As the diagnosis of APS was confirmed, anticoagulation therapy with warfarin was initiated. It is important to note that at that time, contrary to current recommendations, severe pre-eclampsia alone, without delivery before the thirty-fourth week of gestation, was not part of the clinical criteria for APS. According to the new classification criteria, by 2023 ACR/EULAR [5], the patient scored 15 points (3 points from clinical domains and 12 points from laboratory domains).

**Figure 2:**
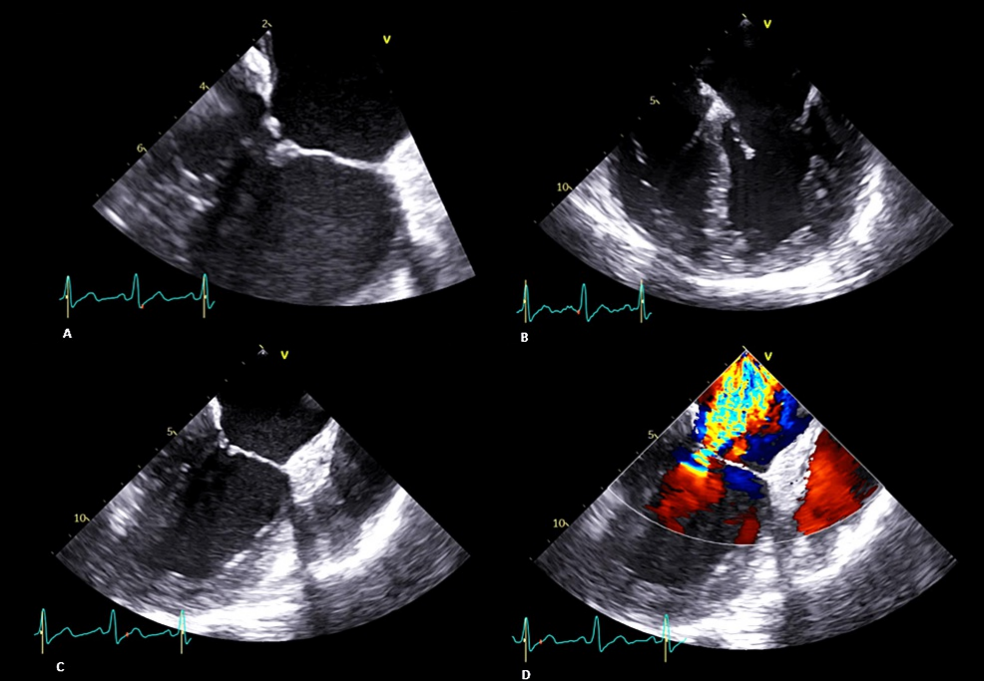
Transesophageal echocardiogram images A, B, and C: Thickened distal ends of the mitral valve leaflets; D: Moderate to severe mitral regurgitation

In outpatient follow-up, the patient's blood pressure remained consistent with second-grade hypertension, and antihypertensive therapy with an angiotensin receptor blocker was initiated. Transthoracic echocardiogram showed severe left atrial dilation, mild left ventricular dilation, with normal wall thickness, and morphological changes in the mitral valve, leading to moderate to severe mitral valve insufficiency, without signs of pulmonary hypertension. To assess the mechanism and severity of mitral insufficiency, a transesophageal echocardiogram was performed, revealing moderate to severe regurgitation due to systolic restriction of the valve leaflets (type IIIb according to Carpentier's classification). It also revealed thickened distal ends of the leaflets with nodular and warty structures measuring 4x4 mm in A2 and P2. The valve's structural changes were considered secondary to Libman-Sacks endocarditis (Figure 2). The patient had no fever or constitutional symptoms and blood cultures were negative, so infective endocarditis was ruled out.

The patient was referred to a cardiology consultation and performed cardiac magnetic resonance that showed a slightly dilated left ventricle, with global systolic function at the lower limit of normality (ejection fraction of 55%) and primary mitral regurgitation (Type IIIa according to Carpentier's classification) quantified as moderate. The right ventricle was not dilated, with normal global systolic function, and no areas of inflammation, fibrosis, or myocardial infiltration were found. A coronary computed tomography angiography showed no coronary atherosclerotic disease and the 24-hour electrocardiogram monitoring also did not show any abnormalities.

Three months later, laboratory assessments indicated an elevated sedimentation rate and positive antinuclear antibodies with a titer of 1/320 with a speckled pattern (AC-5). The panel for antibodies against extractable nuclear antigens and dsDNA antibodies was negative, and complement components 3 and 4 were within the normal range. The creatinine level was 1.14 mg/dL and 24-hour proteinuria was 502.5 mg.

At this moment, the patient scored 10 points on the European League Against Rheumatism (EULAR)/American College of Rheumatology (ACR) 2019 score for SLE, meeting two clinical criteria (4 points regarding acute cutaneous lupus erythematosus, 4 points regarding proteinuria, and 2 points regarding antiphospholipid antibodies). The patient was started on daily 400 mg of hydroxychloroquine. During subsequent blood controls, the patient exhibited a hemoglobin level of 10.9 g/dL, with iron deficiency, and a white blood cell count of 3.9 x 103/uL. Complement component 3 slightly decreased to 77 mg/dL (the lower normal limit is 82). The creatinine level remained stable. Oral iron supplementation was introduced.

Over the last 18 months, the patient kept on Internal Medicine and Cardiology consultations. Throughout this period, she remained asymptomatic, free of cardiovascular complaints, arthralgia, or other symptoms. Physical examination revealed controlled blood pressure and a systolic murmur of grade II/VI was detected at the mitral focus. The most recent blood analysis did not show any cytopenia. The protein/creatinine ratio was 0.18 mg/mg, and creatinine remained stable at 1.1 mg/dL. Additionally, the dsDNA antibody level remained within the reference range (<27 IU/mL). The transthoracic echocardiogram remained the same, with moderate to severe mitral valve insufficiency.

## Discussion

APS is a systemic autoimmune disorder characterized by arterial, venous, or microvascular thrombosis, pregnancy-related morbidity, and nonthrombotic manifestations in individuals with persistent positive antiphospholipid antibodies. The systemic autoimmune disease most frequently associated with APS is SLE [3-5].

The diagnosis of SLE relies on its initial presentation with a characteristic constellation of signs and symptoms, supported by concordant serological analysis, and the exclusion of differential diagnosis. Due to the diversity of clinical manifestations of SLE, some patients present a combination of symptoms and positive laboratory findings that facilitate the diagnosis while others present with isolated symptoms or uncommon disease features, turning the diagnosis into a truly challenging process [1]. Patients with SLE and concomitant APS represent a complex endotype of the lupus spectrum [2].

We report this case to illustrate the diagnostic challenge posed by SLE, even in contemporary medicine. The dynamic nature of diagnostic criteria and the reliance on non-specific immune markers contribute to the diagnostic challenges in SLE and APS, sometimes leading to delays in identifying these diseases. This patient initially presented with severe pre-eclampsia. The complementary study led to the identification of a high-risk profile of antiphospholipid antibodies. Additional investigations showed clinical and laboratory criteria of APS and revealed the consequences of Libman-Sacks endocarditis, with moderate to severe mitral valve insufficiency. This rare condition, characterized by the deposition of sterile platelet thrombi on heart valves, performs a noninfectious form of endocarditis [6,7]. Only after a few months of follow-up, the patient met the criteria for SLE. This case highlights the asynchrony in the involvement of different organs in SLE, emphasizing the importance of a multidisciplinary approach, an early diagnosis, and a frequent and prolonged follow-up of these patients.

## Conclusions

The diagnosis of SLE continues to be a challenge in modern medicine due to the asynchronous involvement of various organs. SLE is one of the most frequent systemic autoimmune diseases associated with APS. The dynamic nature of diagnostic criteria and the reliance on non-specific immune markers contribute to the diagnostic challenges in SLE and APS. Both diseases are multisystemic and can have rare forms of presentation, such as Libman-Sacks endocarditis, which is associated with significant valvular dysfunction. This emphasizes the importance of a multidisciplinary approach.
